# Monitoring spatiotemporal changes in global change drivers and their effects on semiarid woodlands and forests - fieldwork protocol

**DOI:** 10.12688/openreseurope.18564.1

**Published:** 2024-12-19

**Authors:** Cristina Branquinho, Bernardo Rocha, Sami Ullah, Maria Alexandra Oliveira, Elena Vanguelova, Helena C. Serrano, Alice Nunes, Adriana Principe, Pedro Pinho, Silvana Munzi, Juliana Monteiro, Rocío Alonso, Mana Gharun, Rossella Guerrieri

**Affiliations:** 1 cE3c - Centre for Ecology, Evolution and Environmental Changes & CHANGE – Global Change and Sustainability Institute, Faculdade de Ciências, Universidade de Lisboa, Campo Grande, Lisbon, Portugal; 2 School of Geography Earth and Environmental Sciences, University of Birmingham, Birmingham, England, UK; 3 Alice Holt Lodge, Forest Research, Farnham, UK; 4 Centro Interuniversitário de História das Ciências e da Tecnologia Faculdade de Ciências, Universidade de Lisboa, Lisbon, Lisbon, Portugal; 5 Ecotoxicolgy of Air Pollution, Centro de Investigaciones Energeticas Medioambientales y Tecnologicas, Madrid, Community of Madrid, Spain; 6 Institute of Landscape Ecology, University of Münster, Münster, Germany; 7 DISTAL, Alma Mater Studiorum, University of Bologna, Bologna, Emilia-Romagna, Italy

**Keywords:** ecosystem health, sampling methods, soil, lichens, vegetation, training school, COST Action, Mediterranean ecosystem

## Abstract

Training schools play a vital role in COST actions, particularly for young researchers, as they provide opportunities to visit international laboratories and learn new methodologies. In May 2024, CLEANFOREST organized its first training school, *Monitoring Spatiotemporal Changes in Global Change Drivers and Their Effects on Semiarid Woodlands and Forests*, held at the Faculty of Science of the Universidade de Lisboa.

The training school included a field trip designed to explore global change drivers and their impacts on semiarid woodlands and forests. Participants engaged in hands-on activities to understand how forest ecosystems interact with key global change factors such as air quality and climate change. They were introduced to various monitoring techniques and parameters for assessing forest health, including ecosystem fluxes, tree physiology, mortality, and regeneration. Additionally, participants examined plant biodiversity and functional ecology, focusing on lichens and their connection to air quality. Soil-related topics, such as the characterization of forest soils and their physico-chemical properties, were also addressed.

Participants applied these methodologies in real-world scenarios, conducting measurements in different grazing management settings to assess their effects on tree growth, biodiversity, and soil properties. They then processed and analyzed the collected data, synthesizing their results to develop recommendations for sustainable forest management in the context of global change.

This paper presents the field trip protocol used at Companhia das Lezírias, where simplified versions of established methodologies for sampling various ecosystem components were employed. The protocol provides a valuable reference for replicating similar studies, ensuring consistency in methodologies for future training and monitoring efforts.

## Plain Language Summary

Training schools provide young researchers, especially PhD students, with opportunities to learn new skills by working with experts. In May 2024, CLEANFOREST held its first training school, *Monitoring Spatiotemporal Changes in Global Change Drivers and Their Effects on Semiarid Woodlands and Forests*, at the Universidade de Lisboa.

Participants went on a field trip to study how environmental changes, like air quality and climate change, impact forests. They learned methods to monitor forest health, looking at tree growth, soil quality, and biodiversity. The training also included studying organisms like lichens, which reflect air quality, and how different grazing practices affect ecosystems.

Using these experiences, participants analyzed data and suggested ways to manage forests sustainably in response to global environmental challenges.

This paper explains the step-by-step approach from the field trip, offering a guide for future studies.

## Introduction

### CLEANFOREST COST Action CA21138

The ability of forests to mitigate climate change hinges on their capacity to adapt to global change drivers like frequent climate extremes and changes in atmospheric pollutants (e.g., carbon dioxide, reactive nitrogen, and sulfur compounds). These drivers can synergistically, antagonistically, or predisposing affect forest ecosystem health, but they are often studied in isolation. This fragmented approach leads to uncoordinated and scattered information across different research communities. Without a holistic view, the future of Europe’s forests and their climate mitigation potential could be misjudged.

CLEANFOREST ( https://cleanforest.eu) is a COST Action started in 2022 that aims to establish an inclusive, multidisciplinary pan-European network that leverages existing expertise and infrastructures (e.g., monitoring networks, manipulation experiments) to coordinate research efforts, standardize measurements, and foster collaboration. This network will advance scientific knowledge, identify research gaps, and provide insights for future experiments and monitoring. Additionally, CLEANFOREST will engage key stakeholders, including policymakers, small companies, and citizen associations, to promote synergies and develop evidence-based solutions to policy, societal, and technological challenges.

### Framework and objectives of the training school

Among the networking opportunities available to COST actions, training schools are particularly important for young researchers, as they allow PhD researchers to visit internationally recognized laboratories and learn new methodologies to apply in their work. In this context, CLEANFOREST organized its first training school, “Monitoring Spatiotemporal Changes in Global Change Drivers and Their Effects on Semiarid Woodlands and Forests,” in May 2024 at the Faculty of Science of the Universidade de Lisboa.

The objectives of the field trip of the training school included a comprehensive exploration of various global change drivers and their impacts on real situations in semiarid woodlands and forests. Through practical work, participants gained an understanding of the interplay between forest ecosystems and key global change factors such as air quality and climate change. They learnt about different monitoring methods and parameters used to assess forest ecosystem health through ecosystem fluxes, tree physiology, mortality, and regeneration. Additionally, participants delved into the biodiversity and functional ecology of plants, including lichens and their relationship with air quality. The training also covered soil-related aspects, including the characterization of forest soils and their physico-chemical properties. Participants applied their knowledge in real-world scenarios, conducting measurements and assessments in different grazing management settings to understand the effects on tree growth, biodiversity, soil decomposition and physico-chemical properties. Finally, participants engaged in data processing and analysis, synthesizing their findings to draw conclusions and recommendations for sustainable forest management in the context of global change. Here, we present the protocol used in the field trip at *Companhia das Lezírias*. In this protocol, the trainers describe simplified versions of well-established methodologies to sample different components of the ecosystem. This document therefore provides a valuable reference for replicating similar studies and applying consistent methodologies in future training activities and monitoring efforts.

## Methods

### Study area

*Companhia das Lezírias* ( https://cl.pt/en/), located in Portugal close to Lisbon, is one of the country's most prominent agricultural enterprises and land management companies. Situated in the heart of the Tagus River estuary, it encompasses over 18,000 hectares (approximately 44,000 acres) of diverse landscapes, including farmland, forests, wetlands, and natural habitats. The company traces its origins back to the 19th century when it was established to promote agricultural development and land reclamation in the region. Over time, it has evolved into a multifaceted organization involved in various agricultural activities, forestry, nature conservation, and ecotourism initiatives. *Companhia das Lezírias* benefits from its strategic location, enjoying a Mediterranean climate characterized by hot, dry summers and mild, wet winters. The proximity to the Atlantic Ocean moderate temperatures and influences weather patterns, creating favorable conditions for agriculture and supporting rich biodiversity.

Agriculture is at the core of *Companhia das Lezírias*' activities, with the cultivation of crops such as cereals, vineyards for wine production and olive groves for olive oil. Livestock farming, including cattle and horses, also plays a significant role in the company's operations. In addition to agriculture, *Companhia das Lezírias* is committed to sustainable land management practices and nature conservation. It oversees the preservation of natural habitats and wildlife, including the protection of endangered species and the restoration of wetlands. The company actively promotes ecotourism initiatives, allowing visitors to experience the region's natural beauty while raising awareness about environmental conservation such as the case of EVOA (Espaço de Visitação e Observação de Aves, https://evoa.pt/)

In conclusion, *Companhia das Lezírias* stands as a testament to the intricate interplay between geology, climate, land use, and history. Its story is one of resilience, innovation, and stewardship, reflecting the enduring connection between humanity and the land we inhabit. Its extensive land holdings, diverse ecosystems, and dedication to responsible stewardship make it a key player in shaping the socio-economic and environmental landscape of the region.

***Climate***

*Companhia das Lezírias* is located at (38° 47′ 24.01 N; 8° 54′ 11.10 W) 10−20 m above sea level and on a gentle 1.6% slope. Long-term mean annual rainfall in the sampling location is 683 mm with a mean annual temperature of 16.07 °C and 831 mm mean annual potential evapotranspiration, using the Thornsthwaite approach (E-Obs ECA&D, 1950−2012). It is representative of the typical Mediterranean climate characterized by hot and dry summers and wet and cold winters as described in Köppen’s classification. Rainfall occurs predominantly from October to April, while summer water deficit prevails from June to September. This geographic area of Portugal is considered a semi-arid region ( Kurz-Besson *et al.*, 2016). Temperature presents minimum values in January and maximum in August. In this region, the monthly mean maximum temperatures have an intra-annual range of 12 to 18 °C in January and 25 to 36 °C in August; whilst the minimum monthly mean temperature in January fluctuates between 3 to 9 °C and 13 to 19 °C in August. The maximum monthly daily precipitation mean occurs in December with circa 4 mm/day and the minimum occurs during the summer months, with almost no precipitation ( Kurz-Besson *et al.*, 2016).

***Geology***

The geology of *Companhia das Lezírias* is characterized by a variety of sedimentary formations that have been shaped over millions of years. Located in the Tagus River estuary, the region's geological features play a significant role in shaping its landscapes, soils, and agricultural potential. Sedimentary rocks are predominant in the geology of *Companhia das Lezírias*. These rocks are formed by the accumulation and compression of sediments over time. Examples of sedimentary rocks found in the area include sandstone and clay. The proximity to the Tagus River estuary has led to the deposition of alluvial sediments in the region. Over millennia, the river has transported sediments from upstream sources, depositing them in the floodplain and delta areas. These river deposits contribute to the fertility of the soil, making them ideal for agriculture. The entire watershed is located on deep Miocene sands and the main soil types include regosols and podzols. The geological composition of *Companhia das Lezírias* has a direct influence on soil formation processes. The combination of alluvial deposits, coastal sediments, and weathered rock materials gives rise to a diverse range of soil types, each with its own characteristics and agricultural potential. These geological features contribute to the region's diverse landscapes, fertile soils, and agricultural productivity, making it a key factor in shaping the socio-economic and environmental dynamics of the area ( Gonçalves *et al.*, 2012; von Essen *et al.*, 2019).

***Land-use***

For over a century, this area was managed as an agro-silvo-pastoral system. Today, it functions solely as a silvo-pastoral system, with sheep replaced by cattle. Cork is the primary economic product, harvested every nine years, yielding an average of 986 kg per hectare. The cattle stocking rate averages 0.47 animal units per hectare. Cork oak woodlands are the predominant land use and the main focus of management, covering 6,751 hectares (78% of the forested area). Other land uses include pine and eucalyptus plantations (1,900 hectares), agricultural fields, pastures, and reservoirs ( Gonçalves *et al.*, 2012; von Essen *et al.*, 2019).

***Drivers of Change***

a) Climate change

The drought has been identified as a significant driver of change in ecosystems like the *Companhia das Lezírias*. According to studies, drought exacerbates existing vulnerabilities and stresses the ecosystem, influencing vegetation dynamics, biodiversity, and ecosystem services. In particular, cork oak woodlands, which dominate the area, are highly sensitive to changes in water availability. Extended periods of low precipitation can lead to declines in tree growth, reduced regeneration of cork oaks, and shifts in the understory composition (such as shrub encroachment). Moreover, the added pressure from agricultural activities in the surrounding floodplain, where nitrogen deposition is elevated, can compound the negative impacts of drought. The combination of climatic stress and anthropogenic activities makes the region more vulnerable to long-term ecological changes, affecting both biodiversity and ecosystem functioning ( Kurz-Besson *et al.*, 2016).

b) Air pollution

**Figure 1.**
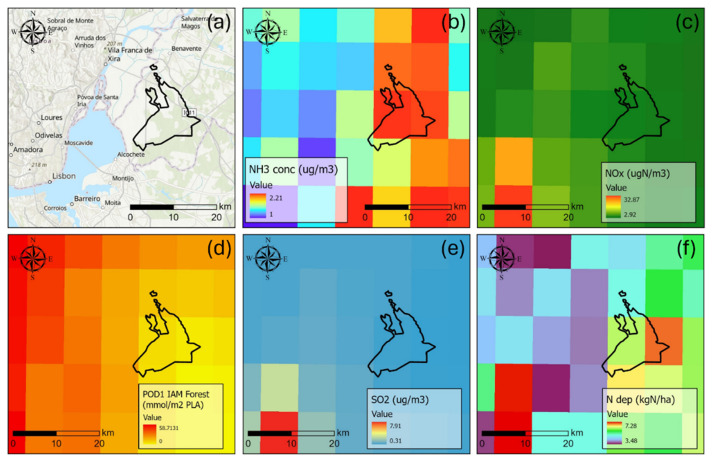
( **a**) Location of the CL estate in the Lisbon area over ArcGISPro world hillshade and topographic map (layer credits: Instituto Geográfico Nacional, Esri, TomTom, Garmin, Foursquare, FAO, METI/NASA, USGS, CGIAR) ; ( **b**) modelled NH _3_ concentration (CLe 1 µg/m ^3^); ( **c**) modelled NO _x_ concentration (CLe 30 µg/m ^3^); ( **d**) modelled POD1IAM for forests (CLe 13.7 mmol/m ^2^ PLA); ( **e**) modelled SO _2_ concentration (CLe 10–20 µg/m ^3^); ( **f**) total nitrogen deposition (CLo ~5 kg/ha). Modelled concentration and deposition from the EMEP MSC-W for 2019 (Data from The Norwegian Meteorological Institute, https://www.met.no/en).

The primary sources of air pollution in the region stem from urban emissions originating in the metropolitan Lisbon area, approximately 45 km to the west, and agricultural activities dispersed across the Tagus floodplain ( Figure 1). Locally, fertilized annual crops, including rice fields and irrigated farmland, surround *Companhia das Lezírias* to the west and northeast, contributing to external nitrogen pollution pressures. Volatilized fertilizers from these high-intensity agricultural zones are carried by prevailing winds into the woodlands located downwind.

Modelled average nitrogen concentrations indicate that critical levels are exceeded only for reduced nitrogen forms (NH _3_), with annual concentrations surpassing the general threshold of 1 µg/m³ for sensitive organisms like lichens and bryophytes. For other pollutants, critical levels (CLe) for vegetation are not exceeded within the study area ( Mills *et al.*, 2017; Pinho *et al.*, 2012). Regarding nitrogen deposition, the recorded levels slightly exceed the indicative lower critical load (CLo) of 5 kg/ha for the *montado* ecosystem ( Bobbink *et al.*, 2022).

c) Grazing

The study area includes extensive cattle farming, where grazing is managed by dividing the land into fenced patches using barbed wire. Grazing levels vary across patches, ranging from areas completely excluded from grazing since 1998 to those with progressively higher cattle densities, with an average of 9.8 cattle heads per hectare per year, a minimum of 19, and a maximum exceeding 300 cattle heads per hectare per year. Grazing intensity stratification was performed using these density values. A grazing intensity index for each patch was calculated based on the number of cattle heads (C) present per plot during each farming year, defined as October to September, over the period from 2007 to 2016.


$$\begin{array}{l} \;Index=[\frac{1}{9}\times C_{2007-2008}+\frac{1}{8}\times C_{2008-2009}+\frac{1}{7}\times C_{2009-2010}+\frac{1}{6}\times C_{2010-2011}+\\ \frac{1}{5}\times C_{2011-2012}+\frac{1}{5}\times C_{2012-2013}+\frac{1}{4}\times C_{2013-2014}+\frac{1}{3}\times C_{2014-2014}+\frac{1}{2}\times C_{2015-2016}\\ \;-(number\;of\;years\;under\;grazing\;exclusion) \end{array}$$

(Where C represents the number of cattle heads per hectare in a given year). The increasing importance attributed to the recent years was used to give extra weight to recent years’ grazing intensity while subtracting the grazing years intended to differentiate sites with grazing exclusion for many years.

d) Tree decline

**Figure 2.**
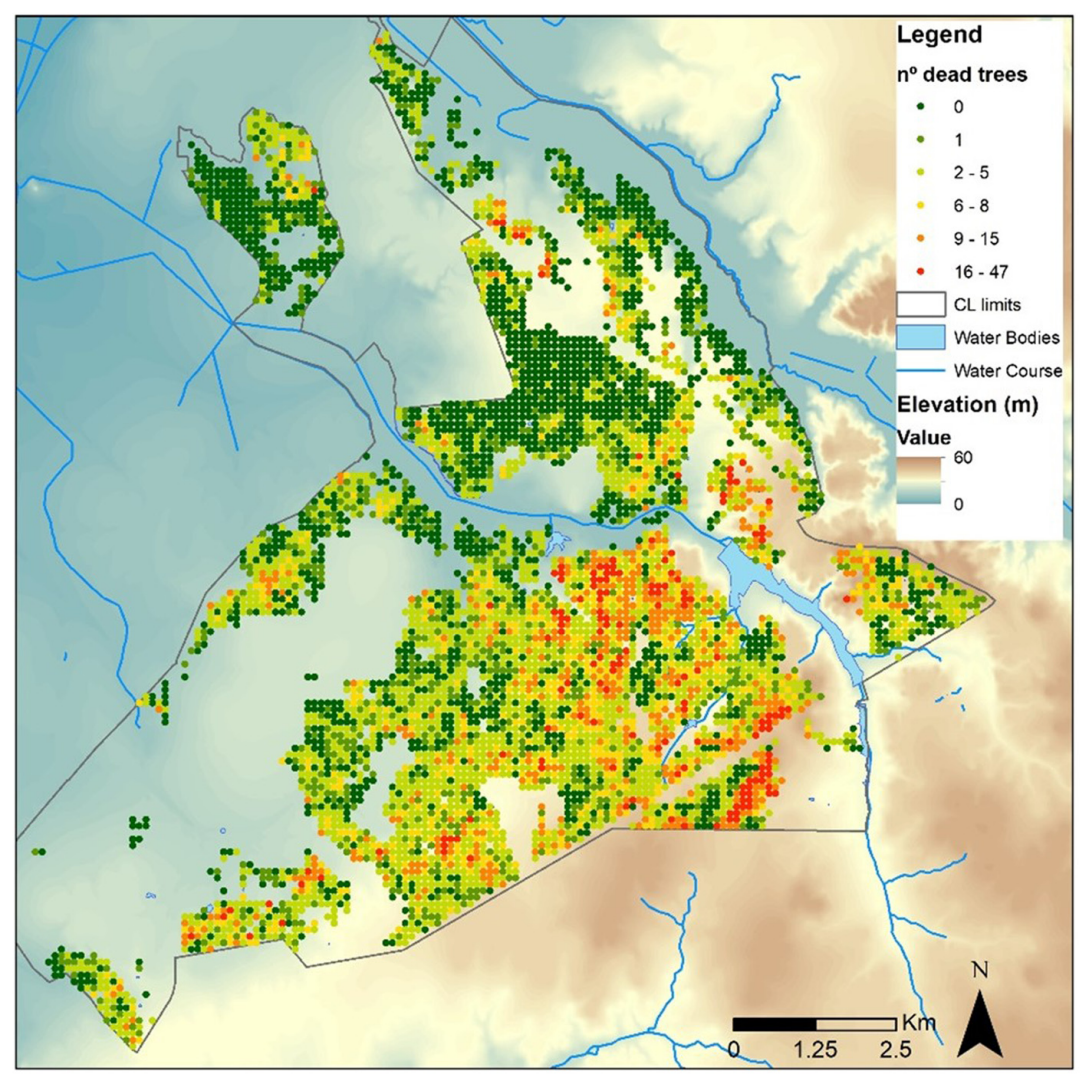
Cork oak tree mortality (n⍜ dead trees/ha) from 2014 to 2019 at *Companhia das Lezírias*. This figure has been reproduced with permission from Príncipe, 2022.

Tree mortality or decline in *Companhia das Lezírias* ( Figure 2) could be influenced by various factors, including:

i) Drought and water stress: in regions with Mediterranean climate, like Portugal, droughts can be a significant stressor for trees, leading to reduced water availability and increased susceptibility to diseases and pests.
ii) Soil degradation: poor soil quality, erosion, or soil compaction can affect tree health and growth, leading to mortality or decline over time.
iii) Climate change: climate change can exacerbate existing stressors on trees, such as increasing temperatures, altered precipitation patterns, and more frequent extreme weather events, all of which can negatively impact tree health and increase mortality rates.
iv) Land use management changes: changes in land use practices, such as frequent soil mobilization that might affect trees' lateral roots.
v) Pests and diseases: certain pests and diseases, such as bark beetles or fungal pathogens, can attack trees, causing mortality or decline, especially if the trees are weakened by other stressors like drought or poor soil conditions.
vi) Air pollution: pollution from agricultural activities, industrial emissions, or traffic can contribute to tree stress and decline, particularly if the trees are exposed to high levels of pollutants over an extended period.

Understanding the specific factors contributing to tree mortality or decline in *Companhia das Lezírias* would require detailed assessments and monitoring of tree health, environmental conditions, and potential stressors in the area. Implementing effective management strategies, such as improved water management, pest and disease control measures, soil conservation practices, and sustainable land use planning, can help mitigate tree mortality and promote healthier forest ecosystems.

Tree decline, especially on podzols, has already been observed at *Companhia das Lezírias*. The natural regeneration of the tree stand is not uniform, and it is lacking in some places due to grazing and shrub clearing. No fires have been reported in the last 20 years. Conflicting interests arise from the livestock grazing component that affects the natural regeneration of cork oak trees. Recently, interventions and adjustments to promote natural regeneration and active afforestation ( *e.g.*, fencing combined with shrub clearing) have been included in the stakeholder´s management plan.

## Monitoring ecosystem health

### Sampling design

**Figure 3.**
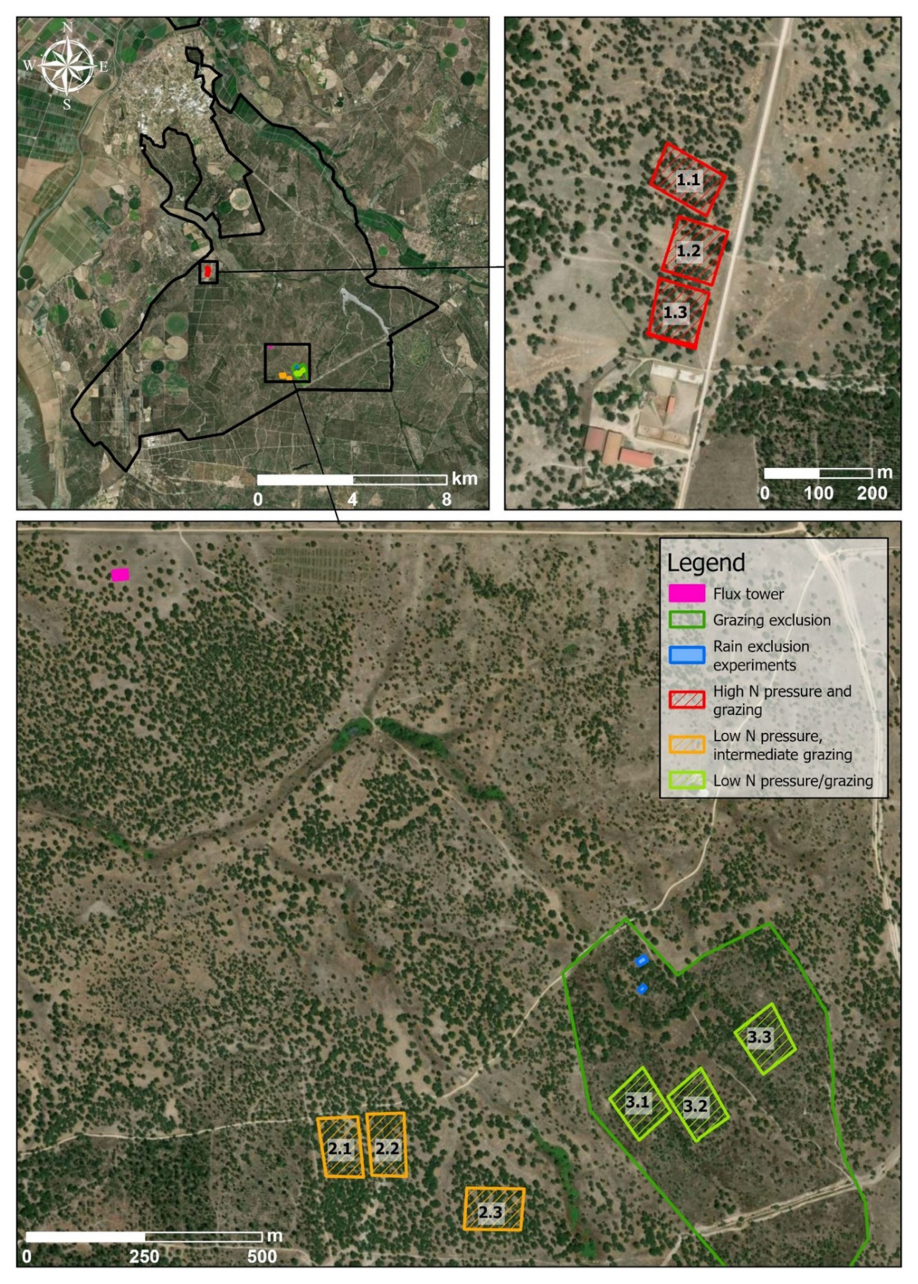
Location of three sampling plots (replicates) within each area (1 - High N pressure and grazing, 2 - low N pressure/grazing and 3 - Grazing exclusion) in *Companhia das Lezírias*. The location of the flux tower and of rain exclusion experiments is also indicated on the map. Ortophoto from ArcGISPro World Imagery layer (Credits: Maxar, Microsoft, Earthstar Geographics).

Select areas with different characteristics in terms, for example, of land use, pollution load, anthropogenic activities, etc. In all areas, several metrics should be measured. In our activity, three areas, corresponding to treatments related to land-use intensity, were sampled: high nitrogen (very high cattle pressure), grazing (low-medium cattle pressure) and control (grazing exclusion) ( Figure 3). Inside each area, 3 plots of 1 hectare (replicates) with at least 30% tree cover and grasses closest to the patch centroid were sampled ( Figure 3). At each plot, the students were divided in groups and assessed: #1 forest structure and lichen diversity; #2 shrub diversity; #3 herbaceous diversity; after assessing all previous indicators all subgroups together sampled #4 soil. Sampling methods for each component are described below.

### Sampling forest structure

Inside a plot with 25-meter radius, measure tree height, and diameter at the breast height (DBH) of all the adult trees (> 10 cm DBH) within the limits of the sampling plot. Most of the trees present inside the plot in our study were cork oaks, then the debarking year and the width of the cork layer were registered.

Select the closest trees in four different quadrants to collect 5 leaves of different ages from one apical branch randomly collected in the south-facing aspects at the same canopy location in each tree. Measure the photosynthetically active radiation and infrared leaf reflectance in each leaf, immediately after cutting the branches, using a UniSpec spectroradiometer (PP-Systems, Haver hills, MA, USA). The reflectance spectra must be assessed in the range of visible light (402 nm) to nearinfrared (1148 nm) wavelengths.

Use three linear belts with 2 m width, 1 m along both sides of linear transects 10 m long, to search for saplings and young trees (the same transects will be used to sample Shrub Diversity). For each young tree, estimate the height using a measuring stick of 1 meter. For all the trees higher than 50 cm, register the diameter at the base of each tree.

### Sampling lichen’s diversity

The European standard protocol for lichen sampling ( Asta *et al.*, 2002; Cristofolini *et al.*, 2014) has been applied in numerous studies to assess lichen biodiversity across environmental gradients such as air pollution, climate, land-use intensity, and management practices ( Lättman *et al.*, 2014; McCarthy *et al.*, 2009; Munzi *et al.*, 2014). Adopting a standardized approach helps minimize variability and potential biases in the data.

**Figure 4.**
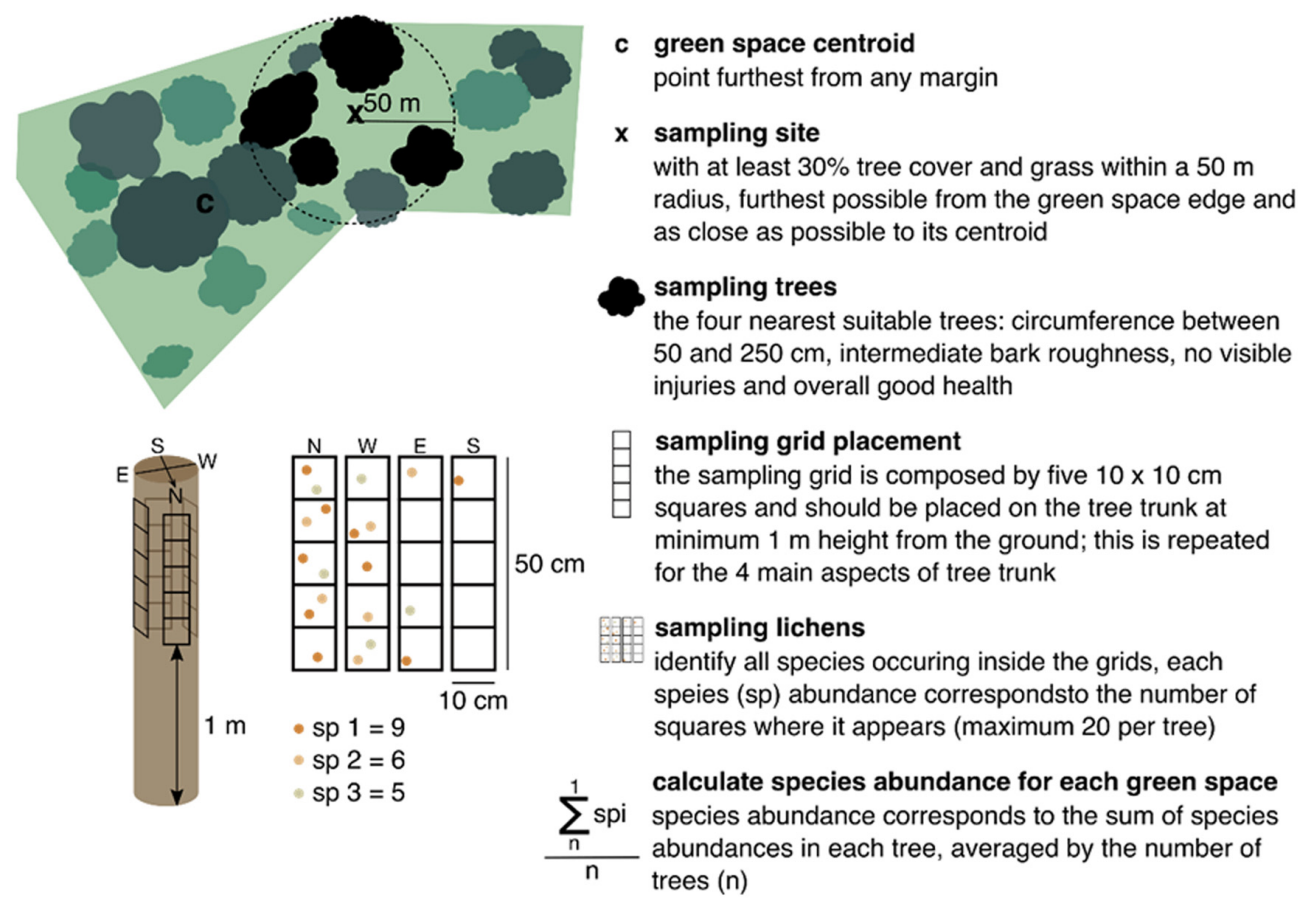
Selection of a single sampling site inside a patch with homogeneous land-cover/use and method to sample lichens diversity, adapted from the European standard method ( Asta *et al.*, 2002; Cristofolini *et al.*, 2014). This figure has been reproduced with permission from Rocha *et al.*, 2022.

At each sampling location, select four trees nearest to the centroid of the site, ensuring they meet the required conditions outlined in the protocol: trees should be healthy, with a straight trunk (no more than a 20° deviation from vertical), free from injuries or branches up to 1.5 meters, and with a circumference at breast height between 50 and 250 cm. The maximum distance between selected trees should not exceed 50 meters ( Figure 4).

To minimize sampling variability, it is crucial to standardize tree characteristics as much as possible. This can be achieved by selecting trees of the same species to maintain consistency in bark texture, pH, and water retention capacity. Place a 50 x 10 cm sampling grid, divided into 5 squares, on each of the four main tree aspects (N, S, E, W), totaling a sampling area of 2000 cm ^2^ per tree ( Figure 4). Record all visible macrolichens (foliose and fruticose) within the grid, either on-site or for later identification in the laboratory. Estimate species abundance based on the number of squares they occupy (with a maximum of 20 occurrences per tree, 5 per aspect), and then calculate the average abundance per site. Species identification should follow the nomenclature guidelines provided by Nimis and Martellos (2021).

### Sampling shrub diversity

**Figure 5.**
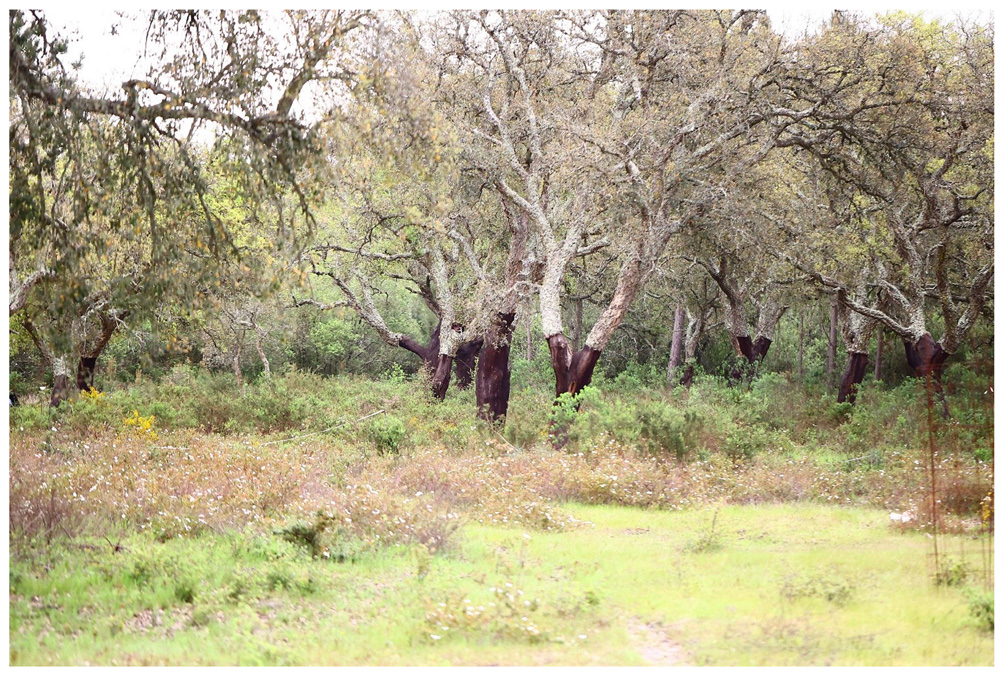
Shrubland patches in the understory of cork oak woodlands ( *montado*).

The understory of cork oak woodlands consists of a mix of shrub-dominated patches embedded within a grassland matrix ( Figure 5). The extent of these shrubland patches, ranging from dense shrub encroachment to sparse or absent coverage, is influenced by various factors. These include grazing intensity, management practices such as shrub clearing, and environmental variables like climate, topography, and soil characteristics ( Nunes *et al.*, 2019).

Although dense shrub cover may be considered not suitable for grazing, shrub communities provide significant ecosystem services. They contribute to carbon storage, enhance soil fertility, and increase nitrogen mineralization rates, potentially supporting ecosystem health and promoting tree regeneration. These benefits depend on the diversity of shrub species and their functional traits (e.g., Eldridge *et al.*, 2024; Köbel *et al.*, 2021).

To sample the shrub community, the line intercept method ( Elzinga *et al.*, 2001) can be applied. This involves placing three 10-meter transects in each plot and measuring the proportion of each species intercepting the transects. Shrub cover is expressed as a percentage based on the intercepted length. Each recorded species is subsequently classified into functional groups for further data analysis.

### Sampling herbaceous diversity

**Figure 6.**
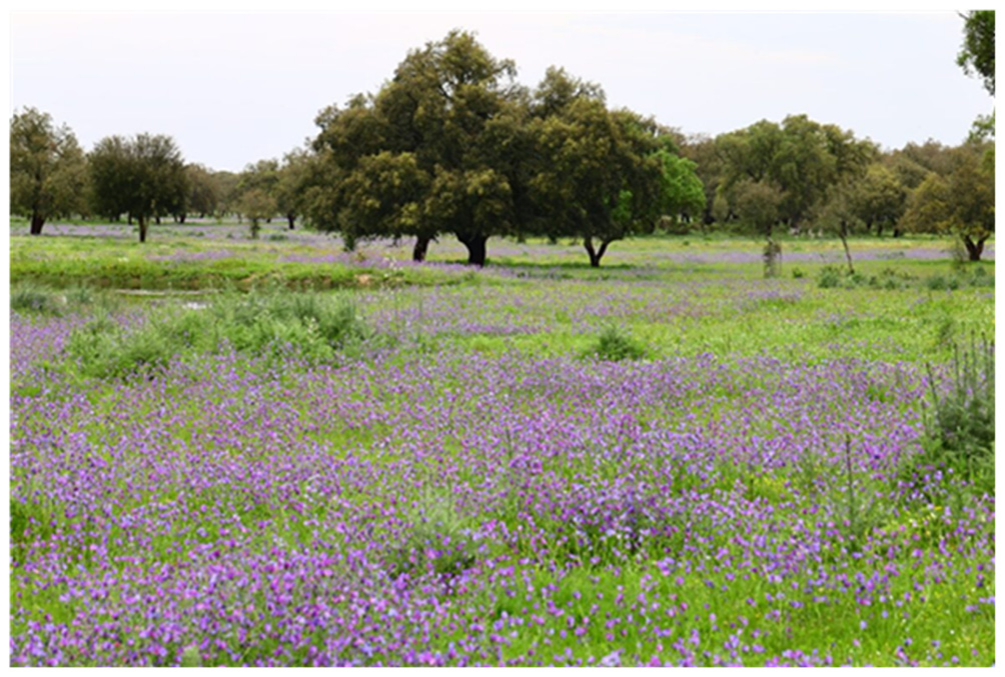
Semi-natural grasslands dominating the understory of cork oak woodlands.

The dominant matrix in the understory of cork oak woodlands is made of semi-natural or sown grasslands, used as pastures ( Figure 6). These communities, dominated by annual herbaceous species, have a remarkably high plant diversity and high resilience to climate interannual fluctuations, characteristic of these ecosystems, showing a faster species turnover than perennials. They play a critical role in ecosystem functioning ( *e.g*., energy flow and nutrient cycling), providing important ecosystem services (e.g. quantity and quality of pasture for livestock, protection against soil erosion), which largely depend on the functional traits of the dominant species.

**Figure 7.**
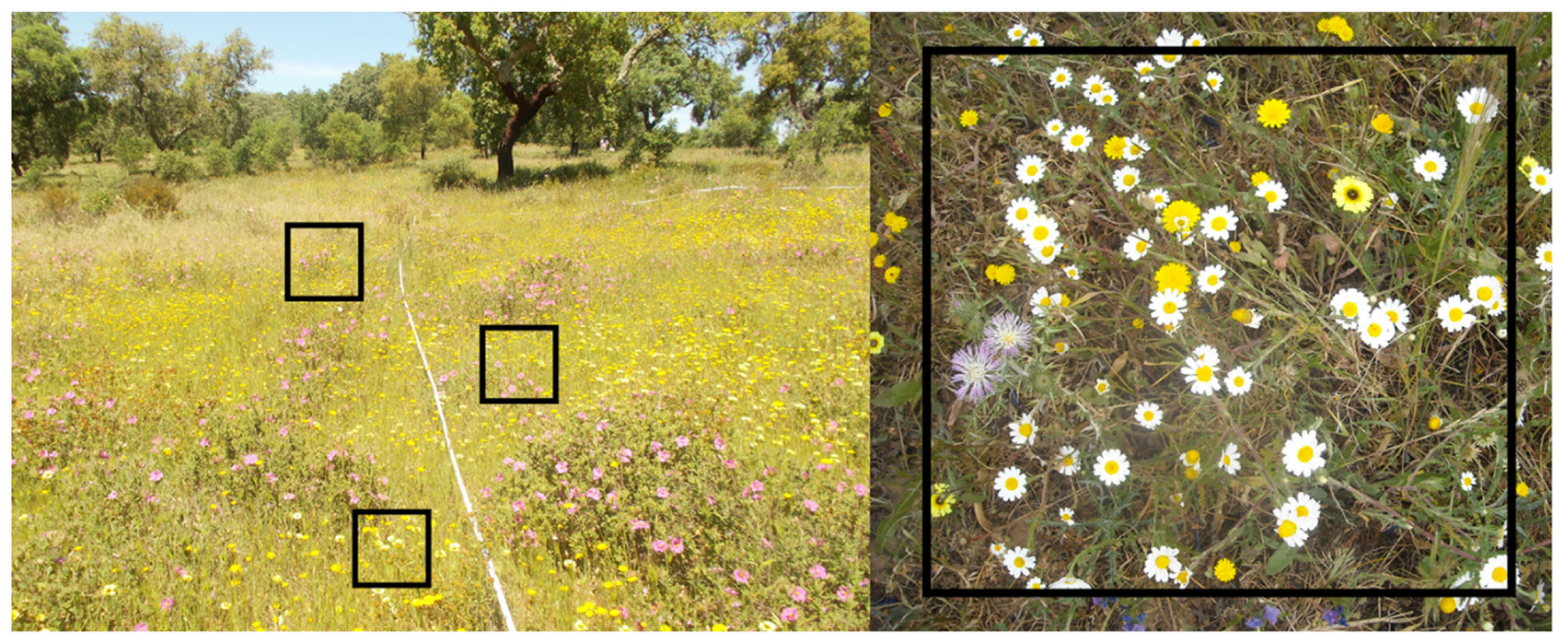
Sampling scheme to measure herbaceous community diversity, with three 50 x 50 cm quadrats placed along alternate sides of a 10 m transect.

Sampling of the herbaceous plant community in each plot can be done using quadrats of 50 x 50 cm, estimating the percentage of the area covered by each species or functional group (e.g. grasses vs. forbs) ( Figure 7). Place three quadrats on alternate sites along three 10 m transects, totalling nine quadrats in each plot.

### Measurements of forest deadwood

Forest deadwood plays a vital role in forest ecosystems, offering habitat, nutrients, and shelter to a wide variety of organisms. It serves as a recognized indicator of forest biodiversity, reflecting habitat quality, ecosystem health, and structural diversity within forests.

Assessing forest deadwood involves measuring several components, including fallen dead trees, coarse woody debris (CWD), snags, and stumps. Coarse woody debris is defined as deadwood with a diameter greater than 10 cm and is typically assessed through full sampling within a known plot area, such as a circular plot with a 10-meter diameter. Fine woody debris, characterized by diameters smaller than 10 cm, can also be measured using a similar approach.

For fine woody debris specifically, pieces with diameters between 5 cm and 10 cm are recorded by measuring both their diameter and length. This ensures a comprehensive understanding of the forest’s deadwood composition across different size classes.

**Coarse woody debris** (CWD) refers to stems, limbs, and branches lying on the ground within the plot. The length of a CWD piece is measured only if its diameter is at least 10 cm. For pieces with irregular diameters along their length, only the sections with a diameter of 10 cm or more are considered, excluding parts below this threshold. Diameter measurements are taken at the midpoint of the segment where the diameter exceeds 10 cm.

**Stumps** are classified as deadwood with a height (or length, if lying) of less than 130 cm from the base and a diameter of at least 10 cm. The stump diameter is measured at the standard cut height.

A **snag** refers to **standing dead wood** taller than 130 cm with a diameter of at least 10 cm. If the snag retains branches, it is categorized as a standing dead tree, and measurements are taken for diameter at breast height (DBH), at 130 cm from the base. The height of the snag is measured up to the point where its diameter decreases to 10 cm. If the snag is shorter than 130 cm, it is treated as a stump.

Decay stages are recorded using a five-class system based on Hunter (1990), to document the degree of decomposition.

**The forest deadwood measurements include:**

•  Diameter (in cm) of coarse woody debris (>10 cm diameter) and length (in m)

•  Diameter of stump (cm) less than 130 cm in height with a diameter at normal cut height greater than 10 cm

•  Estimated diameter of snag (cm) and snag height (in m)

•  Decay state (5 classes) of all deadwoods

Record all measurements in a notebook including **types of deadwoods** (lying, standing, stumps), **length/height (m) and diameter (cm)** and **Declay class** (1 to 5).

### Soils under forest ecosystems

Soil is a mixture of solids (organic and inorganic), water, air and micro-organisms. The components of the soil interact and affect each other and the study of the physical, chemical, hydrological and biogeochemical properties of soils is called soil science. A desirable composition of soils for plant growth is about 25% air, 25% water and ~50% solid material inclusive of organic matter as source of nutrients for plants and carbon for microbial activities. Soil supports plants and provides water and nutrients to them.

Different physico-chemical processes of soil formation lead to transformation and leaching of the soil materials across the soil's depth, which finally results in the formation of distinctive horizontal soil layers (horizons) ( https://en.m.wikipedia.org/wiki/File:Soil_Horizons_Diagram.jpg). Horizons vary in thickness although the boundaries are not always easy to demarcate. Some of the main soil horizons/layers follow:

- **Organic/Humus (O) Layers/Horizons:** ( **OL** – Litter layer; **OF** – Fermentation layer; **OH** – Humification layer) The **OL** horizon is characterized by the accumulation of mainly leaves/needles, twigs and woody materials, most of the original plant organs being easily discernible to the naked eye. The **OF** horizon is characterized by the accumulation of partly decomposed litter, mainly from transformed leaves/needles, twigs and woody materials, but without any entire plant organ. The **OH** horizon is characterized by an accumulation of zoogenically transformed material, i.e. black, gray-brown, brown, reddish-brown well-decomposed litter, mainly comprised of aged animal droppings. LFH is also referred to as O-horizon in some classifications and vice versa.
- **A Horizon:** A mineral horizon formed at or near the surface below O/LFH horizon, where mineral material is mixed with organic matter from the O-horizon. This layer shows accumulation of organic matter and loss of minerals (eluviation of Fe, Al and Clay).
- **E Horizon:** A mineral soil horizon formed in some soils where there lots of the mineral and organic acids are eluviated from it leaving a colorless layer between A and B horizons. Most prominent in podzol soils under coniferous/pine forests.
- **B Layer:** Formed under A-horizon through material accumulation eluted from A horizon and/or by the alteration of the parent material. Accumulation of clay, Fe, Al, Si, humus, CaCO _3_, CaSO _4_, or loss of CaCO _3_; or accumulation of sesquioxides is typical of the B horizon.
- **C Layer:** A soil horizon where evidence of pedogenic [soil forming] activity is not prominent.
- **R Layer:** Consolidated bed rock
- **W Layer:** Water Layer

Dug soil pits in woodland soils under grazing and grazing enclosures to assess the impacts of livestock grazing, nutrient enrichment due to livestock faeces, urine, and atmospheric reactive nitrogen deposition on the physico-chemical properties of soils. In addition to the impact assessment, soil descriptions, decomposition (using the teabag method) and field texture classification by hands should be demonstrated on site. These observations allow discussion of the role of soil in ecosystem health, nutrient supply to trees, carbon sequestration, mcirobial functions, regulation of the water cycle and biodiversity support and the potential impact of grazing and pollution on soil properties, functions and ecosystem services delivery.

***Humus type classification***

Description of humus type follows the European Humus Type Classification only down to the five main terrestrial references ( **Mull**, **Moder**, **Amphi**, **Mor** and **Tangel**) which are identified and described by diagnostic features ( Zanella *et al.*, 2011). 

***Soil horizons***

**Figure 8.**
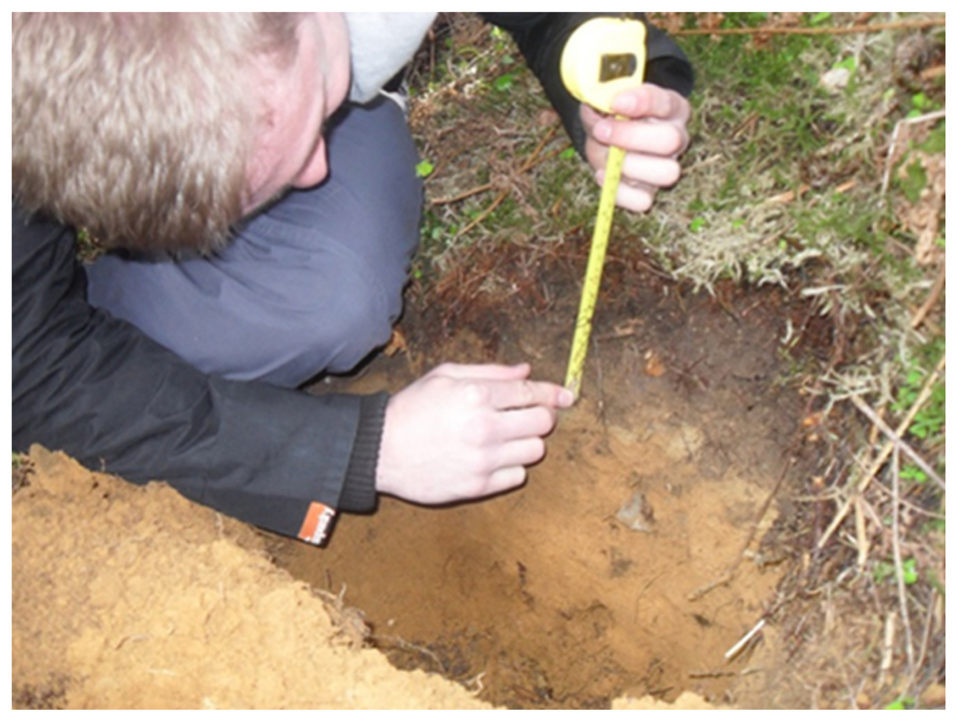
Soil pit (image source: S. Ullah, University of Birmingham).

Description of soil horizons is critical whenever you undertake site characterisation for various purposes such as soil carbon and GHG, fertility studies, remediation of contaminated soils, restoration of ecosystems and waste recycling. Soil sampling for biogeochemical properties is also influenced by horizons and thus it is critical to consider soils horizons while sampling. Protocols for soil horizons follow:

- A soil pit (50 cm wide and 100 cm deep) will be dug to observe and record soil horizons ( Figure 8).
- Try to minimize impacts on the site.
- Identify soil horizons in the pit and record it in your field notebook.
- Record the organic layers presents (LFH) and their depths.
- Record the depth of each horizon in cm starting from 0 cm at the mineral soil surface.
- Record the fragment content in the soil profile.

***Soil colour***

Soil color is important for soil classification, comparison and identifying influence of water saturation, organic matter accumulation, plant-soil interactions and biogeochemical processes in soil formation, and diagnostic colour development. Determine the hue, value and chroma of each soil horizon using the Munsell soil colour charts (2010).

- Hue: Dominant colour of soil (yellow, red, blue, etc.)
- Value: Degree of lightness or darkness (values from 2 to 6, where 2 is the darkest and 6 the lightest).
- Chroma: Purity/strength of colour or degree of colour saturation. Soils have 5 levels of chroma where the lower levels are dull (washed) while the higher levels are purer.

***Soil texture***

Soil texture represents the proportion (percent by weight) of sand, silt, and clay in soil. Soil texture influences soil water retention, permeability, aeration, and retention of nutrients. The texture can be estimated in the field qualitatively by hand. When moist soil is manipulated in the hand, depending on the relative proportion of the particle sizes in it (sand, silt, and clay), the soil feels different. Protocols for texture characterization follow:

- Take a sample of soil after removing litter, organic matter, and stones into the palm of the hand.
- Knead and moisten the soil (where necessary) to make a soil ball in your palm.
- Use the ribboning method supplied to determine texture (printed protocols).
- Report the texture in your report for your presentation.

***Rock fragments***

The presence of rock fragments influences the nutrient status, permeability, use and management of the soil; the accurate calculation of carbon and nutrient stocks; it also reflects its origin and stage of development. Large fragments (>2 mm) are described according to abundance, size, shape, state of weathering and nature of the fragments. The abundance classes correspond with those for surface coarse fragments and mineral nodules. Abundance (in volume %) of rock fragments in each horizon will be estimated using the visual charts ( FAO, 2006) and recorded in field notebook.

- 0 None 0 %
- 1 Very few to few 0 – 5 %
- 2 Common 5 – 15 %
- 3 Many 15 – 40 %
- 4 Abundant 40 – 80 %
- 5 Dominant >80 %

***Root’s abundance***

The presence or absence of roots is the most crucial data to record. If there is a relevant change in the quantity or size of the roots, it is important to provide an explanation for this variation. Potential factors that could limit root growth include compaction (which can be assessed through bulk density measurements), cementation, or a discontinuous pore system.

A qualitative description of root size and abundance is also essential. In some cases, noting additional details, such as abrupt changes in root orientation, may be helpful. Root abundance should only be compared within the same size class. For fine and very fine roots, their abundance can be recorded similarly to voids, expressed as the number of roots per decimetre square.

**Size (diameter)**

- VF - Very fine < 0.5 mm
- F - Fine 0.5 – 2 mm
- M - Medium 2 – 5 mm
- C - Coarse > 5 mm

**Abundance (number of roots/dm ^3^)**

**<2mm root diameter**
  • N - None 0
  • V - Very few 1 – 20
  • F - Few 20 – 50
  • C - Common 50 – 200
  • M - Many >200

**>2mm root diameter**
  • N - None 0
  • V - Very few 1 – 2
  • F - Few 2 – 5
  • C - Common 5 – 20
  • M - Many >20

***Rooting depth***

Record the depth in the soil profile (in cm) where still visible roots are present. The effective rooting depth may be defined as the depth of the soil at which root growth is strongly inhibited. Rooting depth being plant specific, it is recommended that representative species are used to indicate the effective rooting depth of the soil. The effective rooting depth is governed by such factors as the presence of cemented, toxic or compacted layers, hard rock, or indurated gravel layers. A high permanent water table may also control the rooting depth, but may change after drainage. The effective hydrological depth may be much greater. Apart from obvious situations such as the presence of hard rock, it is realized that the estimation of effective soil depth is subject to individual interpretation.

***Biological features***

Describe krotovinas, termite burrows, insect nests, worm casts, or burrows of larger animals in terms of abundance and kind. Record specific locations, patterns, size, composition or any other characteristic.

**Abundance of biological activity** is recorded in the following general descriptive terms:

- N - None
- C - Common
- F - Few
- M - Many

**Examples of biological features** are the following:

- B - Burrows (unspecified)
- E - Earthworm channels
- BO - Open large burrows
- P - Pedotubules
- BI - Infilled large burrows
- T - Termite or ant channels and nests
- C - Charcoal
- Other insect activity

***Soil volumetric water content and temperature***

Soil pore spaces are often filled partially or fully with water. Soil water is important for plant growth, root and nutrient uptake, nutrient cycling and transport of organic and inorganic materials including toxic substances. Soil temperature is important for soil microbial activity, soil biota and soil mineralization and decomposition processes. Volumetric water content (VWC) represents the total volume of soil occupied by water and is often reported in % units. Measure VWC using a portable soil moisture probe to assess if grazing may have changed soil VWC in the topsoil layers. Measure soil temperature by portable temperature probe.

- Using the soil moisture and temperature probes, carefully insert the probes into the soil near the soil pit vertically in the top 10 cm of the soil.
- Remember, if you hit a rock while inserting the probes, then remove the probe and try to insert it in another nearby spot. Don’t force it down as this can break down the probe.
- Repeat the VWC and soil temperature measurements at 5 locations with a minimum distance of a couple of meters between locations.
- Record the soil VWC (%) and soil temperature (C°) readings in your notebook.

***Soil pH and electrical conductivity***

Soil pH is a key variable affecting varied biogeochemical process dynamics and transformations. Soils with high pH (usually >6) are capable of supporting diverse plant species and possess high potential for nutrient supply than acidic soils. Leaching and absorption of nutrients and heavy metals (As, Cd, Pb etc.) are also influenced by soil pH among other factors and thus pH is an important variable to measure to assess soil health and fertility as a first step.

Electrical conductivity (EC) of soils is a surrogate variable for the assessment of the amount of soluble ions (cations and anions). Excessive EC can be observed in saline and sodic soils (> 5 m Siemens/cm). A very low EC may mean soils may have limited available nutrient ions.

Measurement protocols follow:

- Collect about 10 g soil sample from the O and A horizons into a plastic beaker and add 20 ml of DI water to it.
- Shake it or mix it by slowly shaking the beaker in your hand to produce a slurry.
- Using the soil pH + EC probe, measure the pH and EC of the slurry (supernatant) and record in your field book.
- Make an effort to compare the pH and EC of different soil horizons under grazing and grazing enclosures in your field notebook.

***Soil infiltration capacity***

The ability of soil to absorb surface water (rain, irrigation, etc.) is generally reported in mm per hour or per day. Soil infiltration capacity is important for calculating nutrient and pollutant leaching potential into groundwater and surface run-off generation after rainfall, flooding, or an irrigation event. Compaction of soils due to grazing and agricultural machinery results in the reduction of infiltration capacity and an increase in surface run-off, erosion, and flooding. Forests are generally efficient in ameliorating flooding as they have high infiltration capacity.

An instrument like a Geopack’s ^®^ infiltrometer can be used for calculating the infiltration rates of soils. In this method, a cylinder is inserted into soils (up to 4 cm depth) and is filled with water. The water infiltrates the soil pore spaces and excess water is then transmitted down the soil profiles. Infiltration is initially fast and then becomes constant after the pore spaces are fully saturated with time.

Detailed protocol follows:

- Carefully insert the beveled end of the cylinder into the soil to a depth of 40 mm (4 cm). If you realize that the cylinder is against a rock or hard surface in the soil, then remove it from that spot and try inserting it somewhere else close by. Don’t force it as it could be broken.
- Choose a fall unit (a known volume of water within the cylinder). Usually 1 cm to 5 cm. If it is a wet rainy day, a fall unit of 0.5 to 1 cm would be appropriate.

Once you have chosen the fall unit, fill the cylinder up to the 10 cm mark with water and simultaneously start the timer. Repeat the infiltration measurements till you reach an equilibrium as infiltration is relatively faster initially till it reaches an ***equilibrium*** rate.

- Two to five recordings in your notebook need to be taken when doing this experiment. Record the total elapsed time from the start to the point when the infiltration rate becomes constant and record the time taken for a given volume of water to infiltrate the surface (Infiltration rate).
- Record the time taken for the water level to fall by the chosen fall unit (water volume). Record the time it took for the fall unit to infiltrate in the column and next to it record the amount of water (in mm or cm).
- Quickly re-fill the cylinder to the 10 cm mark and record the time again taken for the water level to fall by the fall unit. Record time and fall unit again.
- Repeat the procedure until the time taken for the fall unit becomes constant.
- Finally record the total elapsed time (from the start of the experiment to the end when fall unit infiltration becomes constant).
- Now calculate Infiltration Rates (IR):
  IR = Fall in water level (mm)/Time taken to fall this amount (seconds)
  IR is then in mm/sec or mm/hour or mm/day as you see it appropriate.

***Soil ammonium and phosphate concentrations***

Key soil macronutrients like nitrogen, phosphorus, calcium, etc. in bioavailable forms are critical for the growth of trees. Whilst forest soils are generally limited by available nitrogen and phosphorus forms, grazing can result in nutrient enrichment with ions like ammonium, nitrate, and phosphate. Thus, we will demonstrate and measure soil ammonium and phosphate concentrations using portable colorimetric devices for ammonium and phosphate ions. In these methods, soil will be extracted with DI water (1:20 ratio of soil to DI water), filtered through 0.45 micron meter filter paper and then a 10 ml filter solution will be poured into cuvettes. The solutions will be treated with compound specific reagents to react with ammonium and phosphate for producing specific stable coloured compounds. The solution will then be put into portable colorimetric devices for the determination of the concentration of ammonium and phosphate. For details about nutrient colorimetry, refer to the US EPA Methods for nutrient analysis in soil and water as well as Sgouridis *et al.* (2023).

***Soil carbonates***

The presence of calcium carbonate (CaCO _3_) can be established in the field by adding some drops of 10% HCl to the soil. Is the matrix calcareous or non-calcareous (the exact quantity on carbonates can be tested in the laboratory) will be determined by if there is a reaction when applying the HCl to the soil. If traces are found in at least one horizon of the profile, the presence/absence should be recorded for all horizons.

***Leaf litter decomposition potential – teabag’s method***

The nutrient cycle in forest soils revolves around the uptake of nutrients for the plant growth, followed by the senescence of leaves back into the soil, their breakdown by physical, chemical and/or by other living organisms (herbivores, macro and micro fauna and flora), in a long process back into their most basic units (mineral nutrients, CO _2_) that can be again assimilated by other living organisms.

One method to evaluate the rate of decomposition of leaf litter in soils, and thus, a relative measure of the soil health (presence of biodiversity and potential to perform the ecosystem service of decomposition) is to use standardized leaf litter bags – teabags – incubated in the soil for a period of time (3 months to 3 years). In the last few years a large number of comparisons was possible around the globe, because one of the main factors that determines the rate of decomposition - the composition of the litter - was standardized, using the same type of Green Tea and Rooibos Tea teabags (URLs: teacomposition.org; teatime4science.org). Using leaf litter composition as a fixed factor, we can focus on the factors related to the abundance of soil decomposing microorganisms (namely fungus and bacteria) and to the soil properties ( *e.g*., pH, texture, C:N ratio, organic matter) or climate of the study site.

Methods Lab I: (1) the teabags are dried (40–60 °C) and weighed previously in the lab. (2) each one is labelled with a number ID that will identify it during the duration of the experiment. (3) At least 10 teabags are used as control (not going to the field), dried, weighed, and separated into tea leaves, and teabag with cord and label, and weighted again, to determine the average weight of the packaging to subtract from the original teabags used in the field. (4) calculate a minimum of 2 teabags per site, but preferably more, to account not only for soil niche diversity, but also the probability of losing the teabags during the exposure time (positive correlation, as more time = more losses) due to animal activity or root overgrowth.

Field methods: (5) place the teabags in the organic soil layer (if it exists) or in the mineral soil layer about 5–10 cm below the surface. Unless it is an agricultural soil already disturbed, try not to disturb the soil too much, so natural underground networks are preserved. If that is not possible, try to make the same level of soil disturbance in every plot. (6) They should be spaced ca. 5–10 cm apart, and in a pattern that facilitates the recovery ( *e.g*., in line). (7) the tea tag should be visible and protected, to facilitate the identification of each teabag, GPS coordinates collected, and preferably some form of visual ID or metal detection tags implanted, to easily identify the location later on. (8) after some time (3 months to 3 years) return to the location to (carefully) collect the teabags, making sure the numbers are discernible. Discard any if outside the soil, or with holes. (9) Remove excess soil from the exterior and bring to the lab to dry as soon as possible.

Lab methods: (10) dry the teabags (40–60 °C). (11) remove the remaining tea leaves from the bags, making sure all of them are removed and that no soil goes with it (when in doubt if soil entered the bag or contaminated the leaves during the opening of the teabags, burn the leaves to calculate their organic matter content). (12) From the ID of each teabag, identify the initial weight of the leaves and compare with this final weight, to calculate the amount decomposed.

% Decomposition = (W_initial-W_final)/W_initial *100

***Organic layer density and soil bulk density determination and soil chemical, physical and biological evaluation***

Possible sampling methods:

- Collection of soil samples from the soil profile for chemical, and physical measurements for soil classification purposes.
- Collection of organic layer samples by using known size quadrat and depth measurements to calculate organic layer mass and density for estimation of soil carbon and nutrient stocks.
- Collection of soil samples for estimation of soil bulk density by using 100 cm ^3^ cylindrical stainless-steel rings horizontally inserted and taken from each horizon from the soil pit.
- Collection of samples for earthworms count and species evaluation by using quadrats.
- Collection of samples for soil microbial, mesofauna, nematodes communities' analysis (e.g. by metabarcoding or other molecular methods).
- Collection of samples for fine roots morphological, biomass, density evaluation using cylindrical cores of known volume.
- Collection of soil sampling by Dutch auger for general chemical analysis.

Different sampling approaches and design to cover spatial and temporal scale soil variability and change are available.

## Data Availability

"No data are associated with this article."
