# Supplementary material for: Monitoring spatiotemporal changes in global change drivers and their effects on semiarid woodlands and forests - fieldwork protocol
Source: Open Res Eur. 2025 Mar 26;4:270. Originally published 2024 Dec 19. [Version 2] doi: 10.12688/openreseurope.18564.2 (PMC11976219; doi:10.12688/openreseurope.18564.2)
Supplement: Peer review history

# Reviewer report: Reviewer response for version 2

**DOI:** 10.21956/openreseurope.21555.r52397

**Published:** 2025-04-07

**Authors:** Francesco Valerio

**Affiliations:** 1 BIOPOLIS, Universidade do Porto Centro de Investigacao em Biodiversidade e Recursos Geneticos, Vairão, Porto, Portugal

The revised manuscript shows a commendable improvement over the earlier version. The introduction now flows much more naturally, offering a clear and explicit description of the study’s aims while effectively linking the fieldwork protocol to the broader context of global change drivers. Also, although the study does not involve direct manipulation experiments, the authors have adeptly incorporated relevant literature to provide a nuanced ecological perspective. This approach not only strengthens the theoretical framework of the study but also situates the research within current environmental debates, making the manuscript more engaging for readers.

 The connection to European policy initiatives, such as the European Green Deal, Nature Restoration Law, and Forest Monitoring Law, is presented in a clear and compelling manner. This explicit linkage underscores the practical implications of the research and highlights its potential impact on forest management practices across Europe. I find that the way the manuscript bridges the gap between scientific inquiry and policy relevance is particularly appealing. The authors have further emphasized that their protocol is built upon established, cost-effective methods, resulting in a practical handbook that is especially valuable for training purposes. Finally, the inclusion of a new limitations section further enhances the transparency of the study by openly discussing potential challenges, such as biases in lichen identification and issues in vegetation sampling and soil classification.

 In light of these substantial improvements, I find the manuscript to be a valuable contribution to the literature on forest ecosystem monitoring under global change drivers, and I recommend its acceptance.

Is the study design appropriate for the research question?

Yes

Is the rationale for, and objectives of, the study clearly described?

Yes

Are sufficient details of the methods provided to allow replication by others?

Yes

Are the datasets clearly presented in a useable and accessible format?

Yes

Reviewer Expertise:

My research areas are Ecology, Landscape Connectivity, Remote Sensing, Conservation Biology, and Natural Resources. This combination reflects an interdisciplinary approach and aligns with studies focusing on environmental monitoring, habitat connectivity, and resource management.

I confirm that I have read this submission and believe that I have an appropriate level of expertise to confirm that it is of an acceptable scientific standard.

# Reviewer report: Reviewer response for version 1

**DOI:** 10.21956/openreseurope.20075.r49308

**Published:** 2025-01-28

**Authors:** Javeriya Hasan

**Affiliations:** 1 Toronto Metropolitan University (formerly Ryerson University), Toronto, Canada

This protocol adequately covers the different details of parameters that need to be investigated in line with the research objective of measuring spatiotemporal changes on semiarid woodlands. 

 It only describes the methods which would need to be carried out in an investigation of this sort.

 I would have hoped to see a flow chart or a decision hierarchy governing the sequence of steps to be taken in such an investigation. Additionally, it would have been useful to present a table, infographic or a visual schematic showing how data relating to the different parameters is collected. An example of a data collection pertaining to say "biological features" or "soil volumetric water content and temperature" in tabular form would have been helpful for the reader.

Is the study design appropriate for the research question?

Partly

Is the rationale for, and objectives of, the study clearly described?

Yes

Are sufficient details of the methods provided to allow replication by others?

Yes

Are the datasets clearly presented in a useable and accessible format?

Partly

Reviewer Expertise:

Resilient buildings, sustainability, solar energy, solar architecture, data analytics, machine learning, building science

I confirm that I have read this submission and believe that I have an appropriate level of expertise to confirm that it is of an acceptable scientific standard.

# Response

**Published:** 2025-03-11

**Authors:** Silvana Munzi

**Affiliations:** Universidade de Lisboa, Lisbon, Lisbon, Portugal

We sincerely thank the reviewer for their constructive time and effort in evaluating our work. We really appreciate the positive evaluation and the valuable suggestions. A decision hierarchy and a visual schematic summarizing the data collection process are now included as suggested by the reviewer.

# Reviewer report: Reviewer response for version 1

**DOI:** 10.21956/openreseurope.20075.r49005

**Published:** 2025-01-07

**Authors:** Francesco Valerio

**Affiliations:** 1 BIOPOLIS, Universidade do Porto Centro de Investigacao em Biodiversidade e Recursos Geneticos, Vairão, Porto, Portugal

Dear authors, your manuscript presents a valuable protocol for monitoring spatiotemporal changes in global change drivers and their effects on semiarid woodlands and forests. The methods and focus on training activities align well with COST Actions and Horizon Europe priorities. However, several areas require improvement to enhance the scientific rigor, contextual relevance, and practical applicability of your work. 

 The absence of data associated with this article possibly weakens its replicability. It might be relevant to include illustrative datasets or simulated examples to demonstrate the protocol’s effectiveness and provide insights into the expected outcomes. Also, some examples with associated metadata might increase the reachability of your study.

 While the introduction discusses the need for holistic approaches to studying global change drivers, it does not specify what the study aims to achieve with this protocol. Even if not mandatory, it is advisable to include defined objectives and research questions to enhance the manuscript's clarity, impact, and alignment with COST's mission. In this direction, this research seems to quantify the impacts of varying grazing intensities, nitrogen deposition, and climatic stressors on tree regeneration, biodiversity, and soil health. Also, another valuable point is the applicability of standardized methodologies across diverse management scenarios to ensure replicability and scalability. The proposed protocol finally aims to provide actionable insights to inform sustainable forest management and align with EU biodiversity and climate objectives.

 Global change drivers (e.g., climate change, nitrogen deposition, grazing) are discussed individually, with no exploration of their interactions. I suggest to introduce a conceptual framework to illustrate the synergies and trade-offs among these drivers. For instance, discuss how nitrogen deposition and grazing jointly influence biodiversity and ecosystem health.

 The manuscript mentions sustainable forest management but does not sufficiently link its findings to EU policy frameworks. Please, expand the discussion to highlight how the protocol aligns with European objectives (e.g., Green Deal), such as climate resilience and biodiversity conservation. Provide actionable recommendations for policymakers and stakeholders.

 There is a need to clarify how the proposed protocols fill possible gaps in existing methodologies. Basically, even without comparisons with existing frameworks, there is a need to know what is the novelty of your framework more explicitly. In this direction, the methods section is comprehensive, but it lacks prioritization, as some protocols are described without justification for their inclusion. Therefore, provide a clear rationale for their selection and relevance.

 Include a section discussing potential limitations of the protocol, such as sampling bias or challenges in adapting methods to other ecosystems. 

 Minor issues:

 "Companhia das Lezírias stands as a testament to the intricate interplay between geology, climate, land use, and history." This is overly poetic and not aligned with the technical focus of the proposed protocol.

 "Most of the trees present inside the plot in our study were cork oaks, then the debarking year and the width of the cork layer were registered." The sentence is unclear in construction and lacks specificity about why the debarking year and cork layer width are critical metrics for the protocol.

 "Participants applied these methodologies in real-world scenarios, conducting measurements in different grazing management settings to assess their effects on tree growth, biodiversity, and soil properties." This general statement is too vague and does not detail the specific methods used in these assessments.

 "Record all visible macrolichens (foliose and fruticose) within the grid, either on-site or for later identification in the laboratory." The option to identify species later in the laboratory could introduce inconsistencies in results if not standardized (e.g., storage or transport conditions).

 "Soil sampling for biogeochemical properties is also influenced by horizons and thus it is critical to consider soils horizons while sampling." This sentence is redundant and could be streamlined for clarity. Additionally, it does not specify how horizons influence sampling strategies.

 "The teabags are dried (40–60 °C) and weighed previously in the lab." The temperature range is broad.

 "Record the hue, value, and chroma of each soil horizon using the Munsell soil colour charts (2010)." While the Munsell system is standard, this statement assumes familiarity with the charts without providing context or resources for those unfamiliar.

 "Detailed protocol follows:" (as an introductory phrase). This phrase appears multiple times without  introducing a genuinely detailed protocol. The description often remains high-level and lacks procedural depth.

 "Participants analyzed data and suggested ways to manage forests sustainably in response to global environmental challenges." This conclusion is overly general and does not specify how these data analyses translated into actionable forest management strategies.

Is the study design appropriate for the research question?

Yes

Is the rationale for, and objectives of, the study clearly described?

Yes

Are sufficient details of the methods provided to allow replication by others?

Yes

Are the datasets clearly presented in a useable and accessible format?

Yes

Reviewer Expertise:

My research areas are Ecology, Landscape Connectivity, Remote Sensing, Conservation Biology, and Natural Resources. This combination reflects an interdisciplinary approach and aligns with studies focusing on environmental monitoring, habitat connectivity, and resource management.

I confirm that I have read this submission and believe that I have an appropriate level of expertise to confirm that it is of an acceptable scientific standard, however I have significant reservations, as outlined above.

# Response

**Published:** 2025-03-11

**Authors:** Silvana Munzi

**Affiliations:** Universidade de Lisboa, Lisbon, Lisbon, Portugal

We sincerely thank the reviewer for their constructive and insightful feedback. Their comments have helped us refine and clarify our manuscript, strengthening its impact and relevance. Here the detailed **answer (A)** to each **comment (C):**

**C: **The absence of data associated with this article possibly weakens its replicability. It might be relevant to include illustrative datasets or simulated examples to demonstrate the protocol’s effectiveness and provide insights into the expected outcomes. Also, some examples with associated metadata might increase the reachability of your study.

**A:** We agree that examples are relevant. Since the methods have been largely employed, there are many published papers that can provide illustrative datasets. We added the following references to some examples present in the literature besides the works already cited in the paper: “For replicability purposes, see studies from Rocha et al,. (2019).” “For the leaf litter decomposition protocol (Teabags) we suggested the reading of many articles published so far related to the TeaComposition initiative (e.g., Djukic et al 2018; Kwon et al. 2021), the TBI index (e.g., Keuskamp et al. 2013; Sarneel et al. 2024), or new experimental tests (e.g., Middelanis et al. 2023). As for local data, those articles mentioned for the TeaComposition Initiative, include results from some of the study sites where the Training School took place.” “For humus type description we followed the EU humus type classification system (Zanella et al., 2011) and for some of the soil properties the protocol reflects on the EU ICP Forest Soil Manual for soil sampling and analysis (Cools and De Vos, 2020). ICP forest monitoring soil data on soil carbon stocks (De Vos et al., 2015) and C:N ratio (Cools et al,2014) has been evaluated at European scale.” “The methods used to assess shrub and herbaceous vegetation cover are commonly used in semiarid woodlands and forests, e.g. see Nunes et al., 2014; Nogueira et al.( 2018) and Köbel et al. (2021, 2023, 2024).      

**C:** While the introduction discusses the need for holistic approaches to studying global change drivers, it does not specify what the study aims to achieve with this protocol. Even if not mandatory, it is advisable to include defined objectives and research questions to enhance the manuscript's clarity, impact, and alignment with COST's mission. In this direction, this research seems to quantify the impacts of varying grazing intensities, nitrogen deposition, and climatic stressors on tree regeneration, biodiversity, and soil health. Also, another valuable point is the applicability of standardized methodologies across diverse management scenarios to ensure replicability and scalability. The proposed protocol finally aims to provide actionable insights to inform sustainable forest management and align with EU biodiversity and climate objectives.

**A:** We agree with the reviewer. To focus more the introduction on the work developed, we have expanded on the objectives of the field trip adding the following text: “Semiarid woodlands and forested ecosystems are increasingly vulnerable to degradation due to multiple environmental drivers, including climate change, land-use change, fragmentation, and pollution. Understanding how different ecosystem components respond to these pressures is essential for assessing overall ecosystem degradation—both in terms of biodiversity and ecosystem functions/services. With this in mind, our goal is to compile and evaluate different methodologies for assessing these impacts, linking each ecosystem component to the drivers it responds to most strongly. Specifically, the field trip conducted as part of the training school aimed to provide participants with hands-on experience in assessing global change drivers and their real-world impacts on semiarid woodlands and forests. Through practical activities, students gained a deeper understanding of how forest ecosystems interact with key stressors such as air quality and climate change. In particular, participants learned to quantify the effects of varying grazing intensities, atmospheric nitrogen pollution, and climatic stressors on multiple aspects of forest ecosystems, including forest structure, tree regeneration, biodiversity, and soil health.”

**C:** Global change drivers (e.g., climate change, nitrogen deposition, grazing) are discussed individually, with no exploration of their interactions. I suggest to introduce a conceptual framework to illustrate the synergies and trade-offs among these drivers. For instance, discuss how nitrogen deposition and grazing jointly influence biodiversity and ecosystem health.

**A:** The complex interactions among global change drivers are the scope of the CLEANFOREST COST Action CA21138, but not of the training school. Moreover, we did not address this from a statistical perspective with the students, as no well-replicated manipulation experiments were available. Instead, our approach to exploring how pollution and climate extremes affect forest structure, soil, and biodiversity was to leverage a gradient of grazing intensity to investigate whether higher nitrogen input from livestock influences ecosystem responses to drought. For clarity, we added: “From the state of the art in the subject, we expect that increased nitrogen deposition can initially enhance plant productivity, potentially benefiting grazers. However, grazing can exacerbate the effects of nitrogen deposition by selectively removing certain plant species, leading to a decline in biodiversity and forage quality. Furthermore, climate change, through increased temperatures and altered precipitation patterns, can further modify these interactions, influencing ecosystem processes and outcomes. These interactions are less known in Mediterranean climate and need further work despite some works having been already developed (e.g. Ochoa-Hueso et al. 2017, Nogueira et al., 2018).”  

**C:** The manuscript mentions sustainable forest management but does not sufficiently link its findings to EU policy frameworks. Please, expand the discussion to highlight how the protocol aligns with European objectives (e.g., Green Deal), such as climate resilience and biodiversity conservation. Provide actionable recommendations for policymakers and stakeholders.

**A: **Thanks for raising this important point. We linked the goals of the training school and results achieved to European forest strategies and regulations within the European Green Deal. In particular, we added the following text in the summary: ‘After the practical experience in the field using these experiences, participants were divided into groups to analyze and discuss collected data together with trainers. Key findings were summarized in presentations, together with main take home messages and suggestions on further questions to be explored and related attributes to monitor. Finally, participants had also the opportunity to link their findings to policy actions, by providing indications on potential sustainable forest management actions to increase resilience of the studied woodland ecosystem, and of mediterranean forests in general, under global change, in line with European strategies and regulations.’ Moreover, the following text was added at the end of the introduction: ‘Finally, participants engaged in data processing and analysis, synthesizing their findings to draw conclusions, suggestions on further questions to explore and recommendations for sustainable forest management to promote biodiversity and increase forest resilience under global change. This way, participants had the opportunity to link scientific advances to forest-related strategies and regulations adopted (e.g., Nature Restoration Law) and under discussion (European Forest monitoring Law and soil strategy) within the European Green Deal.’  

**C: **There is a need to clarify how the proposed protocols fill possible gaps in existing methodologies. Basically, even without comparisons with existing frameworks, there is a need to know what is the novelty of your framework more explicitly. In this direction, the methods section is comprehensive, but it lacks prioritization, as some protocols are described without justification for their inclusion. Therefore, provide a clear rationale for their selection and relevance.

**A:** Our approach does not introduce any novel methodologies but rather utilizes existing and well-established methods that have been successfully applied in similar contexts together to have an holistic perspective on the ecosystem structure and function. The primary aim was to create a handbook that was suitable for participants in our training school and adaptable for other similar initiatives. The methodologies were chosen to maximize the forest aspects considered, avoid the need for costly equipment, and adapt the study to the short duration of the fieldwork. We added the following text to make it clearer: “We focused on presenting these methods in a way that is accessible and practical for users, to have an integrated overview of the ecosystem structure and functioning. The methods were selected for their simplicity, cost-effectiveness (requiring no expensive equipment), and comprehensive coverage of the key aspects of a forest study.”

**C: **Include a section discussing potential limitations of the protocol, such as sampling bias or challenges in adapting methods to other ecosystems.

**A:** Section added as suggested.   “For lichens, a key limitation is the expert knowledge required to accurately identify species. Untrained samplers are likely to overlook microlichens, particularly very small species, introducing a potential bias. Additionally, some species groups, such as Physciaceae or Lepraria, are difficult to distinguish, increasing the risk of misidentifications. Another constraint is that the protocol applies exclusively to epiphytic lichens, making it unsuitable for non-forest areas. The methods used to estimate vegetation cover and diversity are commonly applied in semiarid areas like the one studied. Nevertheless, the number and size of transects or quadrats affect the precision of cover estimates. Their definition relies on a compromise between ensuring that most of the variability in the vegetation is encompassed, which depends also on landscape heterogeneity, and cost-effectiveness in the time and resources needed. A potential limitation for soils is that the protocol does not provide a fully detailed description of soil characterization and classification. However, it refers to key soil classification systems (e.g., WRB, 2020), where comprehensive classification methods are described. The classification of humus types is included at its higher, more practical levels, while more detailed levels are referenced in the EU humus classification guidelines.”   Minor issues:

**C:** "Companhia das Lezírias stands as a testament to the intricate interplay between geology, climate, land use, and history." This is overly poetic and not aligned with the technical focus of the proposed protocol.

**A:** Removed as suggested.

**C: **"Most of the trees present inside the plot in our study were cork oaks, then the debarking year and the width of the cork layer were registered." The sentence is unclear in construction and lacks specificity about why the debarking year and cork layer width are critical metrics for the protocol.

**A: **We removed the sentence and the part of the protocol related to leaf physiology because it was not applied in the training school.  

**C:** "Participants applied these methodologies in real-world scenarios, conducting measurements in different grazing management settings to assess their effects on tree growth, biodiversity, and soil properties." This general statement is too vague and does not detail the specific methods used in these assessments.

**A:** Methods used are now mentioned both in the abstract and the introduction. Namely, we added the text: “(forest structure assessment, lichen diversity sampling, shrub and herbaceous diversity estimation, deadwood measurement and soil physico-chemical analysis).”

**C: **"Record all visible macrolichens (foliose and fruticose) within the grid, either on-site or for later identification in the laboratory." The option to identify species later in the laboratory could introduce inconsistencies in results if not standardized (e.g., storage or transport conditions).

**A: **Many lichen species require microscopic observations or chemical tests and therefore cannot be identified in the field. It is common and safe practice to transport lichens to the laboratory for identification, as they retain their key morphological and anatomical characteristics. For clarity, we added the sentence “Store and transport lichen samples in paper bags, ideally one per sampled tree, to ensure they retain their key morphological and anatomical characteristics needed for identification.”

**C: **"Soil sampling for biogeochemical properties is also influenced by horizons and thus it is critical to consider soils horizons while sampling." This sentence is redundant and could be streamlined for clarity. Additionally, it does not specify how horizons influence sampling strategies.

**A:** We replaced the sentence with a clearer text: “Description of soil horizons is crucial when conducting site characterization for purposes such as soil carbon and GHG assessments, fertility studies, remediation of contaminated soils, ecosystem restoration, and waste recycling. Soil properties — including physical, chemical, and biological characteristics — vary significantly between horizons. Therefore, sampling strategies should account for soil horizons to better explain the variability in the targeted physico-chemical properties. For example, organic horizons typically contain much higher organic matter content than underlying mineral soil horizons, making them a key factor in assessing soil organic matter stocks. Additionally, sampling by horizon is fundamental to underpinning soil classification, as it allows a more accurate description of the soil profile and its development.“

**C:** "The teabags are dried (40–60 °C) and weighed previously in the lab." The temperature range is broad.

**A: **We use 60 °C in our lab, but for others to use, depending on their lab conditions, the range 40-60 °C is acceptable, what will change is the time needed for drying, adjusted accordingly. In the text, we replaced the range for “oven ≤60°C”, as some protocols even mention room temperature as a minimum, although we don’t recommend it due to the possible presence of air humidity that will remain on the samples. Temperatures above ~70°C can also be damaging for the samples, potentially accelerating the decomposition.

**C:** "Record the hue, value, and chroma of each soil horizon using the Munsell soil colour charts (2010)." While the Munsell system is standard, this statement assumes familiarity with the charts without providing context or resources for those unfamiliar.

**A: **We provided a more detailed description and some online resources in the text: “Soil is a complex matrix whose colour is influenced by the soil formation factors including parent material, climate, time, organisms and topography. Therefore, soil color determination is indicative of the soil type and of the prevailing soil management. Determining soil color is thus an important variable in defining and classifying soils. A standard method for soil color determination is based on the Munsell Soil Colour determination ( https://munsell.com/color-blog/defining-soil-profiles/; https://www.floridahealth.gov/environmental-health/onsite-sewage/_documents/act-presentation-03-soil-colors-and-their-interpretation-april-2015.pdf). The soil color is characterized by three properties called hue, value and chroma. Using the Munsell soil colour book, determine these properties as below.”

**C: **"Detailed protocol follows:" (as an introductory phrase). This phrase appears multiple times without  introducing a genuinely detailed protocol. The description often remains high-level and lacks procedural depth.

**A: **We removed the word “detailed”.

**C: **"Participants analyzed data and suggested ways to manage forests sustainably in response to global environmental challenges." This conclusion is overly general and does not specify how these data analyses translated into actionable forest management strategies.

**A: **The sentence is no longer present due to the rearrangement of the text.

# Response

**Published:** 2025-03-22

**Authors:** Silvana Munzi

**Affiliations:** Universidade de Lisboa, Lisbon, Lisbon, Portugal

**C:** The absence of data associated with this article possibly weakens its replicability. It might be relevant to include illustrative datasets or simulated examples to demonstrate the protocol’s effectiveness and provide insights into the expected outcomes. Also, some examples with associated metadata might increase the reachability of your study.

**A:** We agree that examples are relevant. Since the methods have been largely employed, there are many published papers that can provide illustrative datasets. We added the following references to some examples present in the literature besides the works already cited in the paper:   “For replicability purposes, see studies from Rocha et al,. (2019).”   “For the leaf litter decomposition protocol (Teabags) we suggested the reading of many articles published so far related to the TeaComposition initiative (e.g., Djukic et al 2018; Kwon et al. 2021), the TBI index (e.g., Keuskamp et al. 2013; Sarneel et al. 2024), or new experimental tests (e.g., Middelanis et al. 2023). As for local data, those articles mentioned for the TeaComposition Initiative, include results from some of the study sites where the Training School took place.” “For humus type description we followed the EU humus type classification system (Zanella et al., 2011) and for some of the soil properties the protocol reflects on the EU ICP Forest Soil Manual for soil sampling and analysis (Cools and De Vos, 2020). ICP forest monitoring soil data on soil carbon stocks (De Vos et al., 2015) and C:N ratio (Cools et al,2014) has been evaluated at European scale.” “The methods used to assess shrub and herbaceous vegetation cover are commonly used in semiarid woodlands and forests, e.g. see Nunes et al., 2014; Nogueira et al.( 2018) and Köbel et al. (2021, 2023, 2024).

**C:** While the introduction discusses the need for holistic approaches to studying global change drivers, it does not specify what the study aims to achieve with this protocol. Even if not mandatory, it is advisable to include defined objectives and research questions to enhance the manuscript's clarity, impact, and alignment with COST's mission. In this direction, this research seems to quantify the impacts of varying grazing intensities, nitrogen deposition, and climatic stressors on tree regeneration, biodiversity, and soil health. Also, another valuable point is the applicability of standardized methodologies across diverse management scenarios to ensure replicability and scalability. The proposed protocol finally aims to provide actionable insights to inform sustainable forest management and align with EU biodiversity and climate objectives.

**A:** We agree with the reviewer. To focus more the introduction on the work developed, we have expanded on the objectives of the field trip adding the following text: “Semiarid woodlands and forested ecosystems are increasingly vulnerable to degradation due to multiple environmental drivers, including climate change, land-use change, fragmentation, and pollution. Understanding how different ecosystem components respond to these pressures is essential for assessing overall ecosystem degradation—both in terms of biodiversity and ecosystem functions/services. With this in mind, our goal is to compile and evaluate different methodologies for assessing these impacts, linking each ecosystem component to the drivers it responds to most strongly. Specifically, the field trip conducted as part of the training school aimed to provide participants with hands-on experience in assessing global change drivers and their real-world impacts on semiarid woodlands and forests. Through practical activities, students gained a deeper understanding of how forest ecosystems interact with key stressors such as air quality and climate change. In particular, participants learned to quantify the effects of varying grazing intensities, atmospheric nitrogen pollution, and climatic stressors on multiple aspects of forest ecosystems, including forest structure, tree regeneration, biodiversity, and soil health.”

**C: **Global change drivers (e.g., climate change, nitrogen deposition, grazing) are discussed individually, with no exploration of their interactions. I suggest to introduce a conceptual framework to illustrate the synergies and trade-offs among these drivers. For instance, discuss how nitrogen deposition and grazing jointly influence biodiversity and ecosystem health.

**A: **The complex interactions among global change drivers are the scope of the CLEANFOREST COST Action CA21138, but not of the training school. Moreover, we did not address this from a statistical perspective with the students, as no well-replicated manipulation experiments were available. Instead, our approach to exploring how pollution and climate extremes affect forest structure, soil, and biodiversity was to leverage a gradient of grazing intensity to investigate whether higher nitrogen input from livestock influences ecosystem responses to drought. For clarity, we added: “From the state of the art in the subject, we expect that increased nitrogen deposition can initially enhance plant productivity, potentially benefiting grazers. However, grazing can exacerbate the effects of nitrogen deposition by selectively removing certain plant species, leading to a decline in biodiversity and forage quality. Furthermore, climate change, through increased temperatures and altered precipitation patterns, can further modify these interactions, influencing ecosystem processes and outcomes. These interactions are less known in Mediterranean climate and need further work despite some works having been already developed (e.g. Ochoa-Hueso et al. 2017, Nogueira et al., 2018).”  

**C:** The manuscript mentions sustainable forest management but does not sufficiently link its findings to EU policy frameworks. Please, expand the discussion to highlight how the protocol aligns with European objectives (e.g., Green Deal), such as climate resilience and biodiversity conservation. Provide actionable recommendations for policymakers and stakeholders.

**A:** Thanks for raising this important point. We linked the goals of the training school and results achieved to European forest strategies and regulations within the European Green Deal. In particular, we added the following text in the summary: ‘After the practical experience in the field using these experiences, participants were divided into groups to analyze and discuss collected data together with trainers. Key findings were summarized in presentations, together with main take home messages and suggestions on further questions to be explored and related attributes to monitor. Finally, participants had also the opportunity to link their findings to policy actions, by providing indications on potential sustainable forest management actions to increase resilience of the studied woodland ecosystem, and of mediterranean forests in general, under global change, in line with European strategies and regulations.’ Moreover, the following text was added at the end of the introduction: ‘Finally, participants engaged in data processing and analysis, synthesizing their findings to draw conclusions, suggestions on further questions to explore and recommendations for sustainable forest management to promote biodiversity and increase forest resilience under global change. This way, participants had the opportunity to link scientific advances to forest-related strategies and regulations adopted (e.g., Nature Restoration Law) and under discussion (European Forest monitoring Law and soil strategy) within the European Green Deal.’  

**C: **There is a need to clarify how the proposed protocols fill possible gaps in existing methodologies. Basically, even without comparisons with existing frameworks, there is a need to know what is the novelty of your framework more explicitly. In this direction, the methods section is comprehensive, but it lacks prioritization, as some protocols are described without justification for their inclusion. Therefore, provide a clear rationale for their selection and relevance.

**A:** Our approach does not introduce any novel methodologies but rather utilizes existing and well-established methods that have been successfully applied in similar contexts together to have an holistic perspective on the ecosystem structure and function. The primary aim was to create a handbook that was suitable for participants in our training school and adaptable for other similar initiatives. The methodologies were chosen to maximize the forest aspects considered, avoid the need for costly equipment, and adapt the study to the short duration of the fieldwork. We added the following text to make it clearer: “We focused on presenting these methods in a way that is accessible and practical for users, to have an integrated overview of the ecosystem structure and functioning. The methods were selected for their simplicity, cost-effectiveness (requiring no expensive equipment), and comprehensive coverage of the key aspects of a forest study.”

**C:** Include a section discussing potential limitations of the protocol, such as sampling bias or challenges in adapting methods to other ecosystems.

**A:** Section added as suggested.   “For lichens, a key limitation is the expert knowledge required to accurately identify species. Untrained samplers are likely to overlook microlichens, particularly very small species, introducing a potential bias. Additionally, some species groups, such as Physciaceae or Lepraria, are difficult to distinguish, increasing the risk of misidentifications. Another constraint is that the protocol applies exclusively to epiphytic lichens, making it unsuitable for non-forest areas. The methods used to estimate vegetation cover and diversity are commonly applied in semiarid areas like the one studied. Nevertheless, the number and size of transects or quadrats affect the precision of cover estimates. Their definition relies on a compromise between ensuring that most of the variability in the vegetation is encompassed, which depends also on landscape heterogeneity, and cost-effectiveness in the time and resources needed. A potential limitation for soils is that the protocol does not provide a fully detailed description of soil characterization and classification. However, it refers to key soil classification systems (e.g., WRB, 2020), where comprehensive classification methods are described. The classification of humus types is included at its higher, more practical levels, while more detailed levels are referenced in the EU humus classification guidelines.”   Minor issues:

**C:** "Companhia das Lezírias stands as a testament to the intricate interplay between geology, climate, land use, and history." This is overly poetic and not aligned with the technical focus of the proposed protocol.

 A: Removed as suggested.

**C: **"Most of the trees present inside the plot in our study were cork oaks, then the debarking year and the width of the cork layer were registered." The sentence is unclear in construction and lacks specificity about why the debarking year and cork layer width are critical metrics for the protocol.

 A: We removed the sentence and the part of the protocol related to leaf physiology because it was not applied in the training school.  

**C: **"Participants applied these methodologies in real-world scenarios, conducting measurements in different grazing management settings to assess their effects on tree growth, biodiversity, and soil properties." This general statement is too vague and does not detail the specific methods used in these assessments.

**A:** Methods used are now mentioned both in the abstract and the introduction. Namely, we added the text: “(forest structure assessment, lichen diversity sampling, shrub and herbaceous diversity estimation, deadwood measurement and soil physico-chemical analysis).”

**C:** "Record all visible macrolichens (foliose and fruticose) within the grid, either on-site or for later identification in the laboratory." The option to identify species later in the laboratory could introduce inconsistencies in results if not standardized (e.g., storage or transport conditions). **A: **Many lichen species require microscopic observations or chemical tests and therefore cannot be identified in the field. It is common and safe practice to transport lichens to the laboratory for identification, as they retain their key morphological and anatomical characteristics. For clarity, we added the sentence “Store and transport lichen samples in paper bags, ideally one per sampled tree, to ensure they retain their key morphological and anatomical characteristics needed for identification.”

**C:** "Soil sampling for biogeochemical properties is also influenced by horizons and thus it is critical to consider soils horizons while sampling." This sentence is redundant and could be streamlined for clarity. Additionally, it does not specify how horizons influence sampling strategies.

**A:** We replaced the sentence with a clearer text: “Description of soil horizons is crucial when conducting site characterization for purposes such as soil carbon and GHG assessments, fertility studies, remediation of contaminated soils, ecosystem restoration, and waste recycling. Soil properties — including physical, chemical, and biological characteristics — vary significantly between horizons. Therefore, sampling strategies should account for soil horizons to better explain the variability in the targeted physico-chemical properties. For example, organic horizons typically contain much higher organic matter content than underlying mineral soil horizons, making them a key factor in assessing soil organic matter stocks. Additionally, sampling by horizon is fundamental to underpinning soil classification, as it allows a more accurate description of the soil profile and its development.“

**C: **"The teabags are dried (40–60 °C) and weighed previously in the lab." The temperature range is broad.

**A:** We use 60 °C in our lab, but for others to use, depending on their lab conditions, the range 40-60 °C is acceptable, what will change is the time needed for drying, adjusted accordingly. In the text, we replaced the range for “oven ≤60°C”, as some protocols even mention room temperature as a minimum, although we don’t recommend it due to the possible presence of air humidity that will remain on the samples. Temperatures above ~70°C can also be damaging for the samples, potentially accelerating the decomposition.

**C:** "Record the hue, value, and chroma of each soil horizon using the Munsell soil colour charts (2010)." While the Munsell system is standard, this statement assumes familiarity with the charts without providing context or resources for those unfamiliar.

**A:** We provided a more detailed description and some online resources in the text: “Soil is a complex matrix whose colour is influenced by the soil formation factors including parent material, climate, time, organisms and topography. Therefore, soil color determination is indicative of the soil type and of the prevailing soil management. Determining soil color is thus an important variable in defining and classifying soils. A standard method for soil color determination is based on the Munsell Soil Colour determination ( https://munsell.com/color-blog/defining-soil-profiles/; https://www.floridahealth.gov/environmental-health/onsite-sewage/_documents/act-presentation-03-soil-colors-and-their-interpretation-april-2015.pdf). The soil color is characterized by three properties called hue, value and chroma. Using the Munsell soil colour book, determine these properties as below.”

**C:** "Detailed protocol follows:" (as an introductory phrase). This phrase appears multiple times without  introducing a genuinely detailed protocol. The description often remains high-level and lacks procedural depth.

**A:** We removed the word “detailed”.

**C:** "Participants analyzed data and suggested ways to manage forests sustainably in response to global environmental challenges." This conclusion is overly general and does not specify how these data analyses translated into actionable forest management strategies.

**A: **The sentence is no longer present due to the rearrangement of the text.
